# Amelioration of acute liver failure by a cinnamic acid derivative through inhibition of the ROS–NETosis axis

**DOI:** 10.1186/s43556-026-00448-x

**Published:** 2026-04-13

**Authors:** Jie Yin, Longjie Ding, Ziming Zhao, Xia Chen, Jianzheng Huang, Yang Xiao, Lianghu Gu, Xiaotian Zhang, Qingyi Tong, Yonghui Zhang

**Affiliations:** 1 Hubei Key Laboratory of Natural Medicinal Chemistry and Resource Evaluation, School of Pharmacy, Tongji Medical College, Huazhong University of Science and Technology, Wuhan, Hubei China; 2 Institute of Pharmaceutical Process, Hubei Province Key Laboratory of Occupational Hazard Identification and Control, School of Medicine, Wuhan University of Science and Technology, Wuhan, 430065 China

**Keywords:** ALF, ROS, NETosis, Cinnamic acid derivative

## Abstract

Acute liver failure (ALF) represents a critical clinical challenge with limited therapeutic options beyond liver transplantation, urgently requires novel therapeutic strategies. Here, we show that a cinnamic acid derivative, CA7, confers significant protection in mouse models of ALF. The therapeutic benefits of CA7 were evidenced by the preservation of hepatic architecture, reduction of mitochondrial damage and apoptosis, and enhanced survival rates. Using an integrated approach combining single-cell RNA sequencing and functional validation, we found that neutrophils are prominently involved in the hepatic response to CA7 treatment and represent a major cellular population through which CA7 exerts its protective effects. Mechanistically, CA7 exerts its potent hepatoprotective effects by selectively suppressing reactive oxygen species production, thereby inhibiting neutrophil extracellular trap formation, a pivotal trigger of hepatic oxidative injury. This intervention attenuated neutrophil recruitment and activation, restored redox homeostasis, and suppressed activation of the NF-κB–NLRP3–IL-1β inflammatory signaling cascade. As a consequence, CA7 effectively alleviated cytokine storm–associated inflammatory responses, prevented mitochondrial dysfunction and protected hepatocyte from apoptosis. Together, these coordinated actions underlie the robust protective effects of CA7 in ALF. Our findings suggest that CA7 acts as a promising therapeutic candidate for ALF and highlight reactive oxygen species (ROS)-dependent NETosis as an important pathogenic process in life-threatening ALF.

**Supplementary Information:**

The online version contains supplementary material available at 10.1186/s43556-026-00448-x.

## Introduction

ALF is a life-threatening syndrome characterized by rapid hepatic decompensation triggered by diverse etiologies, including drug/chemical toxicity, viral infections, alcoholic liver disease, autoimmune hepatitis, metabolic dysfunction-associated steatohepatitis, or systemic organ failure [1, 2]. Clinically, it manifests as jaundice, coagulopathy, hepatic encephalopathy, ascites, and thrombotic/hemorrhagic complications, often progressing to multiorgan failure with high short-term mortality. Current treatment options are primarily supportive, with liver transplantation remaining a last-resort intervention constrained by significant financial burden and limited donor availability, highlighting the critical need for innovative therapies [1, 3].

ALF pathogenesis is driven by a pathological triad of metabolic dysfunction, oxidative stress, and inflammatory cascades. Excessive reactive oxygen species production and glutathione depletion, serve as a central hub connecting detoxification failure and immune dysregulation. Innate immune hyperactivation further amplifies oxidative stress: Pathogen-Associated Molecular Patterns (PAMPs) and Damage-Associated Molecular Patterns (DAMPs) engage Toll-like receptors (TLRs) on Kupffer cells and neutrophils, triggering pro-inflammatory cytokine release. This creates a self-perpetuating cycle of oxidative damage and inflammation that exacerbates hepatocyte injury [4–7].

Neutrophils play a key role in ALF progression [8–10]. Activated neutrophils release neutrophil extracellular traps (NETs) through neutrophil extracellular trap formation (NETosis), a process distinct from apoptosis and necrosis. NETs normally trap pathogens, but in ALF, dysregulated NET formation—driven by ROS—induces hepatocyte cytotoxicity and amplifies inflammation through TLR activation and cytokine storms, which recruit additional neutrophils and further propagate NETosis. This "ROS–inflammation–NETs" feedback loop represents a critical pathogenic mechanism, suggesting that interventions targeting NETosis, TLR signaling, or pro-inflammatory cytokines may provide new therapeutic avenues [11–17].

Cinnamic acid (CA), a principal bioactive monomer derived from Cinnamomum cassia, and its derivatives exhibit antioxidant, anti-inflammatory, and multi-target pharmacological effects with favorable safety profiles [18–22]. Despite these advantages, their potential in hepatology remains underexplored. In this study, we investigated CA7, a CA derivative, hypothesizing that it mitigates ALF progression by suppressing ROS production and NET formation, thereby reducing hepatocyte oxidative damage, mitochondrial dysfunction, and apoptosis. Using in vivo D-galactosamine/lipopolysaccharide (D/L)–induced acute liver failure models, in vitro neutrophil assays, and single-cell transcriptomics, we demonstrate that CA7 attenuates oxidative stress, inflammation, and NETosis, revealing a novel hepatoprotective mechanism and highlighting its translational potential as a therapeutic agent for ALF.

## Results

### CA7 mitigates D/L-induced ALF and prolongs survival in murine model

**Fig. 1:**
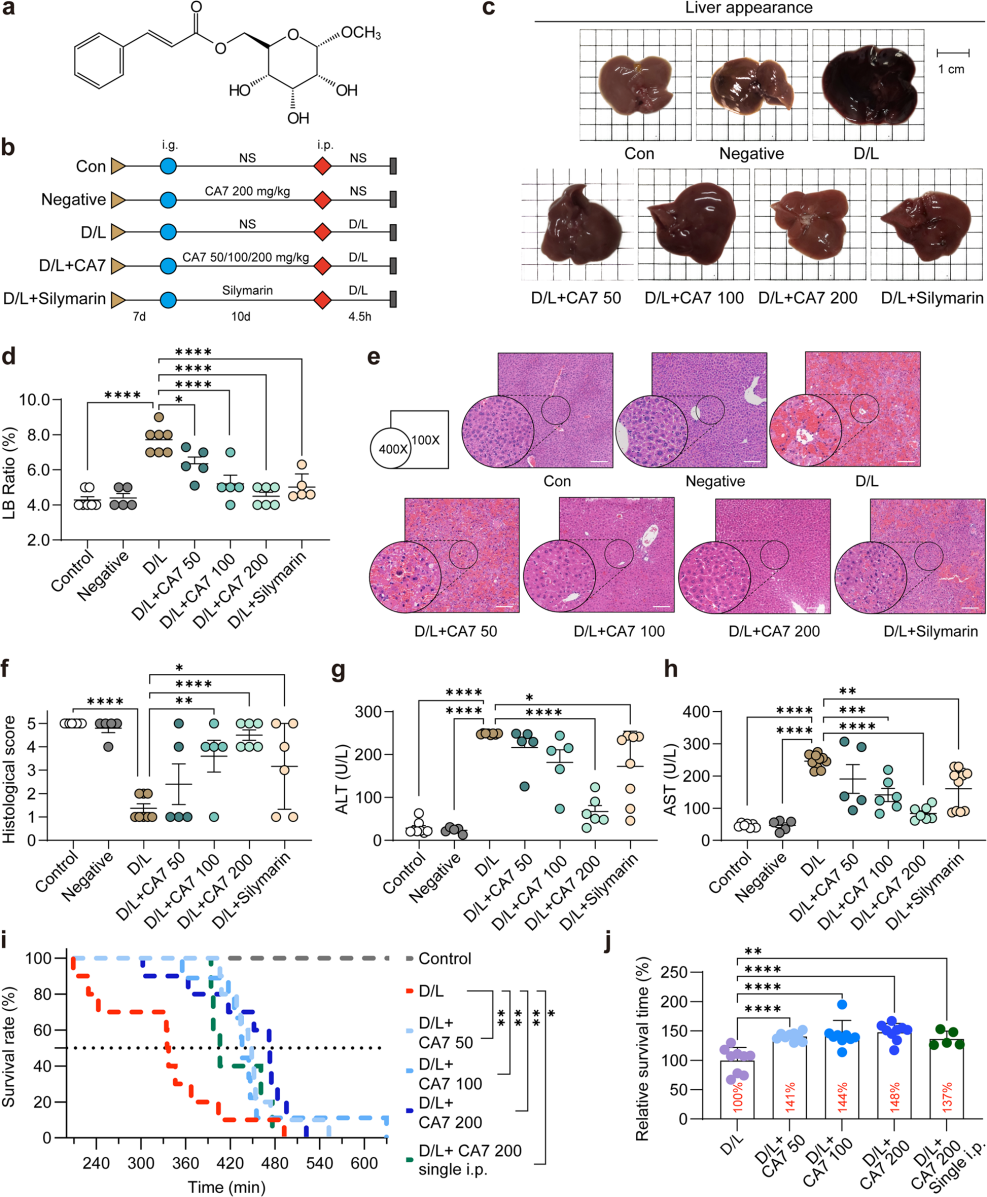
CA7 mitigates D/L-induced ALF and prolongs survival in murine model. **a** Chemical structure of CA7. **b** Schematic representation of the experimental design for the animal study. NS, normal saline. **c** Representative images of mouse livers 4.5 h post-D/L administration. Scale bar: 1 cm. **d** Scatter plot of the LB ratio for each experimental group. *n* = 5–8 samples/group. Data are presented as mean ± standard deviation (SD), and statistical significance was assessed by one-way ANOVA (*p* < 0.05, *p < 0.01, **p < 0.001, ***p < 0.0001; ns, not significant (same notation applies to subsequent figures). **e** Representative H&E-stained liver sections at 400 × and 100 × magnification. Scale bar: 150 μm. **f** Scatter plot of histological scores for H&E-stained liver sections. Mean ± SD. One-way ANOVA. **g** Scatter plot of serum AST levels (*n* = 5–8 samples/group). Mean ± SD. One-way ANOVA. **h** Scatter plot of serum ALT levels (n = 5–8 samples/group). Mean ± SD. One-way ANOVA. **i** Kaplan–Meier survival curve of ALF mice (i.p. *n* = 5; others *n* = 10 samples/group). Survival differences were evaluated using the log-rank (Mantel-Cox) test and Gehan-Breslow-Wilcoxon test. **j** Scatter plot of survival times of ALF mice (*n* = 5–10 samples/group). (i.p. *n* = 5; others *n* = 10 samples/group). Mean ± SD. One-way ANOVA

To identify novel small-molecule compounds with therapeutic potential for ALF, we designed and synthesized a series of cinnamic acid–derived compounds based on the reported bioactivities of cinnamic acid and its derivatives, and systematically evaluated their hepatoprotective effects. Through preliminary screening, CA7 was identified as a lead compound, exhibiting relatively superior hepatoprotective efficacy compared with other derivatives (data not shown). CA7 was subsequently synthesized through a straightforward and cost-effective approach (Fig. 1a, Fig. S1a, b) and further evaluated in a D/L–induced murine model of ALF (Fig. 1b).

As shown in Fig. 1c, treatment with CA7 markedly alleviated hepatic swelling and congestion and substantially improved liver architecture compared with the D/L model group. These effects were comparable to, or exceeded, those observed with silymarin, a standardized extract from milk thistle (Silybum marianum) containing flavonolignans with established antioxidant and anti-inflammatory properties, which was used as a positive control. Consistently, CA7 significantly reduced the liver-to-body weight ratio (LB ratio), further supporting its hepatoprotective activity (Fig. 1d). Hematoxylin and eosin (H&E) staining demonstrated severe hepatic pathology following D/L challenge, including intense inflammatory responses, extensive inflammatory cell infiltration, pronounced hemorrhage, and widespread hepatocyte necrosis—features indicative of fulminant liver failure. In contrast, CA7 treatment markedly attenuated these pathological changes, resulting in substantial reductions in inflammatory infiltration and overall tissue damage, as reflected by significantly improved histological scores (Fig. 1e, f).

Biochemical analyses further corroborated these findings. CA7 treatment led to a marked reduction in serum ALT (alanine aminotransferase) and AST (aspartate aminotransferase) levels, exceeding the protective effect seen in the silymarin-treated group, indicating superior hepatoprotective efficacy of CA7 relative to the positive control (Fig. 1g, h).

Given that rapid progression and high mortality are defining features of ALF, we next assessed the impact of CA7 on survival outcomes. Treatment with CA7 at multiple doses significantly prolonged survival in D/L-induced ALF mice. Notably, survival benefits were observed following both oral administration and a single intraperitoneal (i.p.) injection of CA7 (Fig. 1i). Specifically, oral CA7 administration extended median survival by more than 40%, while intraperitoneal injection achieved a 37% increase in median survival time (Fig. 1j). Collectively, these findings unequivocally demonstrate CA7's potent hepatoprotective effects in D/L-induced ALF, highlighting its potential for further development.

### CA7 alleviates mitochondrial damage and hepatocyte apoptosis in ALF mice

Hepatocyte apoptosis is a critical pathological hallmark in the mouse model of acute liver failure induced by D-galactosamine and lipopolysaccharide [23]. To determine whether CA7 protects hepatocytes by attenuating apoptosis and mitochondrial damage, we next examined liver tissue from ALF mice treated with CA7. As shown in Fig. 1e, histopathological analysis of liver sections revealed pronounced hepatocyte apoptosis in ALF mice, which was markedly ameliorated by CA7 or silymarin treatment, as reflected by significantly improved histological scores compared to the model group.

**Fig. 2:**
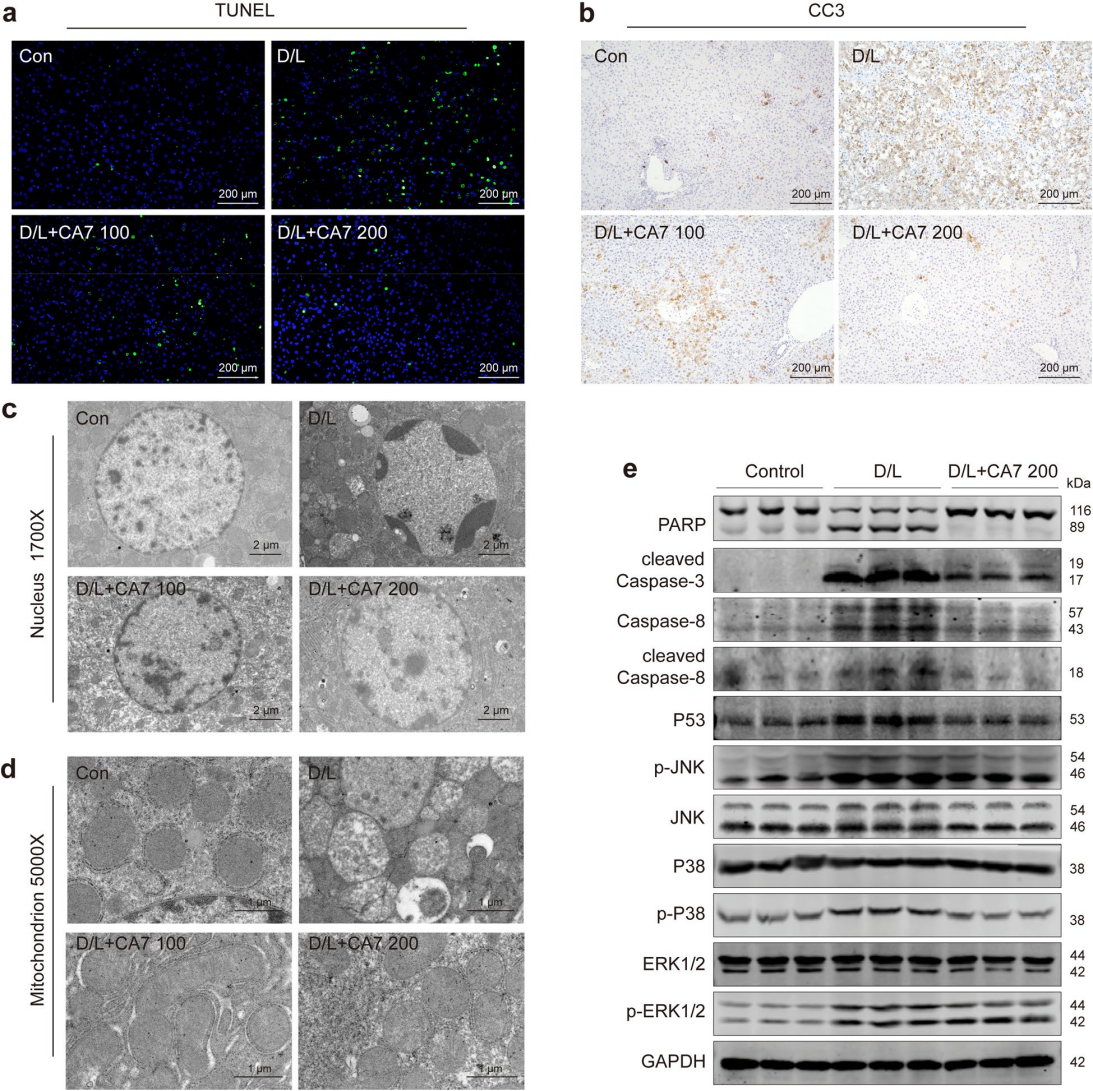
CA7 alleviates mitochondrial damage and hepatocyte apoptosis in ALF mice. **a** Representative image of TUNEL staining in mouse liver sections. Nuclei (blue, DAPI). Scale bar: 200 μm. **b** Immunohistochemical detection of cleaved caspase-3 (CC3) in liver sections from ALF mice. Scale bar: 200 μm. **c** Transmission electron microscopy (TEM) images of hepatocyte nuclei. Scale bar: 2 μm. **d** TEM visualization of hepatocyte mitochondria. Scale bar: 1 μm. **e** Western blot analysis of key apoptosis-related proteins in liver tissues. GAPDH was used as control. *n* = 3 samples/group

Consistent with this, D/L treatment led to severe hepatocyte apoptosis evidenced by the increase of TUNEL-positive cells, which were not observed in CA7 treatment group (Fig. 2a). Consistent with these findings, immunohistochemistry (IHC) analysis further confirmed robust cleaved caspase-3 (CC3) expression in ALF livers, which was dose-dependently suppressed by CA7 (Fig. 2b). Transmission electron microscopy (TEM) revealed classical apoptotic features in the model group, including chromatin condensation and peripheral margination, while CA7-treated hepatocytes maintained uniformly dispersed nuclear chromatin (Fig. 2c). Collectively, these results indicate that CA7 effectively inhibits hepatocyte apoptosis in ALF.

Given the central role of mitochondria in cell death, oxidative stress and inflammatory signaling [24, 25], we next evaluated mitochondrial ultrastructure. As revealed by TEM analysis, ALF induced severe mitochondrial damage, characterized by disrupted inner membrane cristae and increased matrix lucency. In contrast, CA7 treatment markedly preserved mitochondrial morphology, largely reversing these alterations (Fig. 2d). Consistent with these observations, western blot analysis demonstrated that CA7 significantly suppressed the D/L-induced upregulation of key apoptotic mediators, such as cleaved PARP and CC3, p53, and CC8, and suppressed the activation of pro-apoptotic kinases p38 and JNK, kinases critically involved in apoptosis initiation, whereas ERK phosphorylation remained largely unchanged (Fig. 2e). Mechanistically, CA7 did not activate hepatocyte-autonomous pro-survival pathways such as autophagy [26, 27]; instead, it suppressed autophagic activity (Fig. S2), suggesting that its hepatoprotective effects likely arise from modulation of the liver microenvironment rather than direct induction of intrinsic hepatocyte survival programs.

Together, these results demonstrate that CA7 protects hepatocytes in ALF by alleviating mitochondrial damage and suppressing apoptosis.

### CA7 attenuates oxidative stress in ALF mice independently of NRF2 activation

**Fig. 3:**
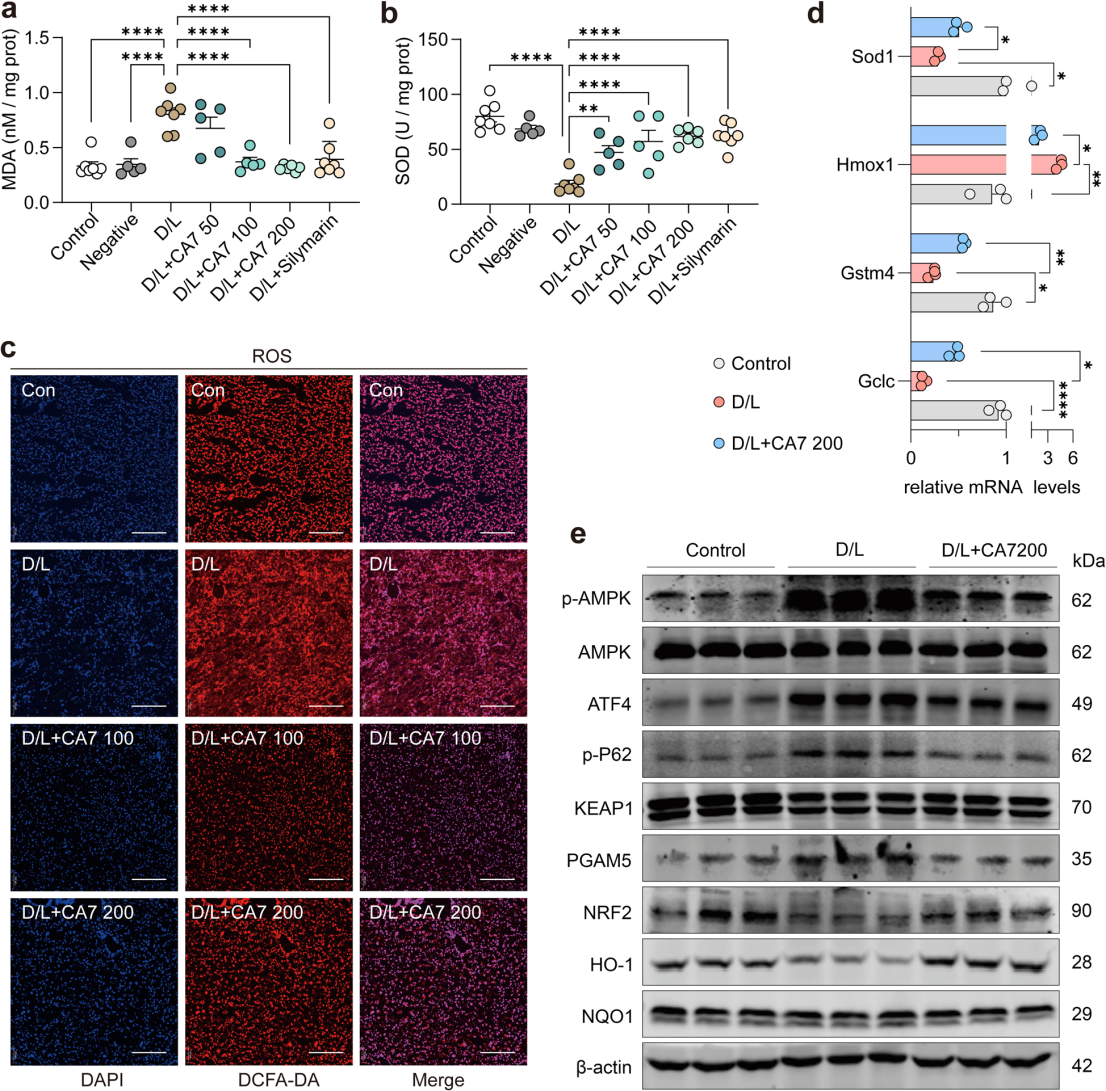
CA7 attenuates oxidative stress in ALF mice independently of NRF2 activation. **a** Levels of MDA in mouse liver homogenates (*n* = 5–7 samples/group). Mean ± SD. One-way ANOVA. **b** SOD activity in mouse liver homogenates (*n* = 5–7 samples/group). Mean ± SD. One-way ANOVA. **c** Representative images of ROS staining in mouse liver tissues, visualized by fluorescent staining; nuclei were counterstained with DAPI (blue). Data are presented as mean ± standard deviation (SD), and statistical significance was assessed by one-way ANOVA. **d** Quantitative real-time PCR (qRT-PCR) analysis of mRNA expression levels for antioxidant-related genes. β-actin was used as control. *n* = 3 samples/group. **e** Western blot analysis of key proteins involved in oxidative stress response and autophagy regulation in liver tissues. β-actin was used as control. *n* = 3 samples/group

Given the close interplay between oxidative stress, inflammation, and hepatocyte injury [6, 28, 29], we next investigated whether CA7 exerts antioxidant effects in D/L–induced ALF mice. Biochemical analyses showed that D/L challenge markedly disrupted hepatic redox homeostasis, as evidenced by reduced activity of the antioxidant enzyme superoxide dismutase (SOD) and increased levels of malondialdehyde (MDA), a lipid peroxidation product. Notably, CA7 treatment significantly restored SOD activity and reduced MDA accumulation, indicating effective attenuation of oxidative stress (Fig. 3a, b). Consistent with these findings, ROS fluorescence staining revealed substantial hepatic ROS accumulation in ALF mice, which was markedly diminished following administration of CA7 (Fig. 3c).

To further characterize the impact of CA7 on oxidative stress–related transcriptional responses, we performed qPCR analysis of key antioxidant and stress-responsive genes. ALF mice exhibited pronounced dysregulation of oxidative stress–associated genes, including downregulation of Gclc (a rate-limiting enzyme in glutathione synthesis), Sod1 (an antioxidant enzyme gene), and Gstm4 (a glutathione S-transferase gene), alongside elevated levels of HO-1 (a marker of intense oxidative stress response). In contrast, these transcriptional alterations were absent in the CA7-treated mice (Fig. 3d). In agreement with the transcriptional data, western blotting revealed that CA7 attenuated the D/L-triggered induction of key oxidative stress signaling molecules, including phosphorylated adenosine 5’-monophosphate-activated protein kinase (AMPK), activating transcription factor 4 (ATF4), and phosphorylated P62, providing molecular evidence that CA7 effectively counteracts oxidative stress in ALF mice (Fig. 3e).

The PGAM5-KEAP1-NRF2 axis is a core regulatory pathway for cellular defense against oxidative stress, in which phosphoglycerate mutase 5 (PGAM5) mediates the mitochondrial recruitment of kelch-like ECH-associated protein 1 (KEAP1), resulting in nuclear factor erythroid 2-related factor 2 (NRF2) dissociation, nuclear import and subsequent activation of antioxidant genes [30, 31]. However, our study found that CA7 did not activate canonical NRF2 signaling: hepatic levels of KEAP1 and NRF2 remained low in the CA7-treated group, comparable to those in the ALF model group. Although PGAM5 was elevated in ALF mice and normalized by CA7 treatment, the NRF2-dependent gene NQO1 failed to be upregulated at both transcript and protein levels. This lack of response supports the notion that CA7 exerts its antioxidant effects via pathways outside the conventional NRF2 axis (Fig. 3e).

Together, these results indicate that CA7 effectively alleviates oxidative stress in ALF mice through an NRF2-independent mechanism, likely acting in concert with its anti-inflammatory and anti-apoptotic effects.

### CA7 suppresses cytokine storm and inflammation for hepatoprotection

Because oxidative stress is a potent upstream driver of inflammatory signaling cascades, including the p38–JNK pathway that we found to be suppressed by CA7 (Fig. 2e), we next investigated whether the therapeutic efficacy of CA7 is associated with suppression of excessive inflammatory responses in ALF [32, 33]. To address this, we performed an unbiased, comprehensive quantification of 31 key cytokines in serum and liver tissues.

**Fig. 4:**
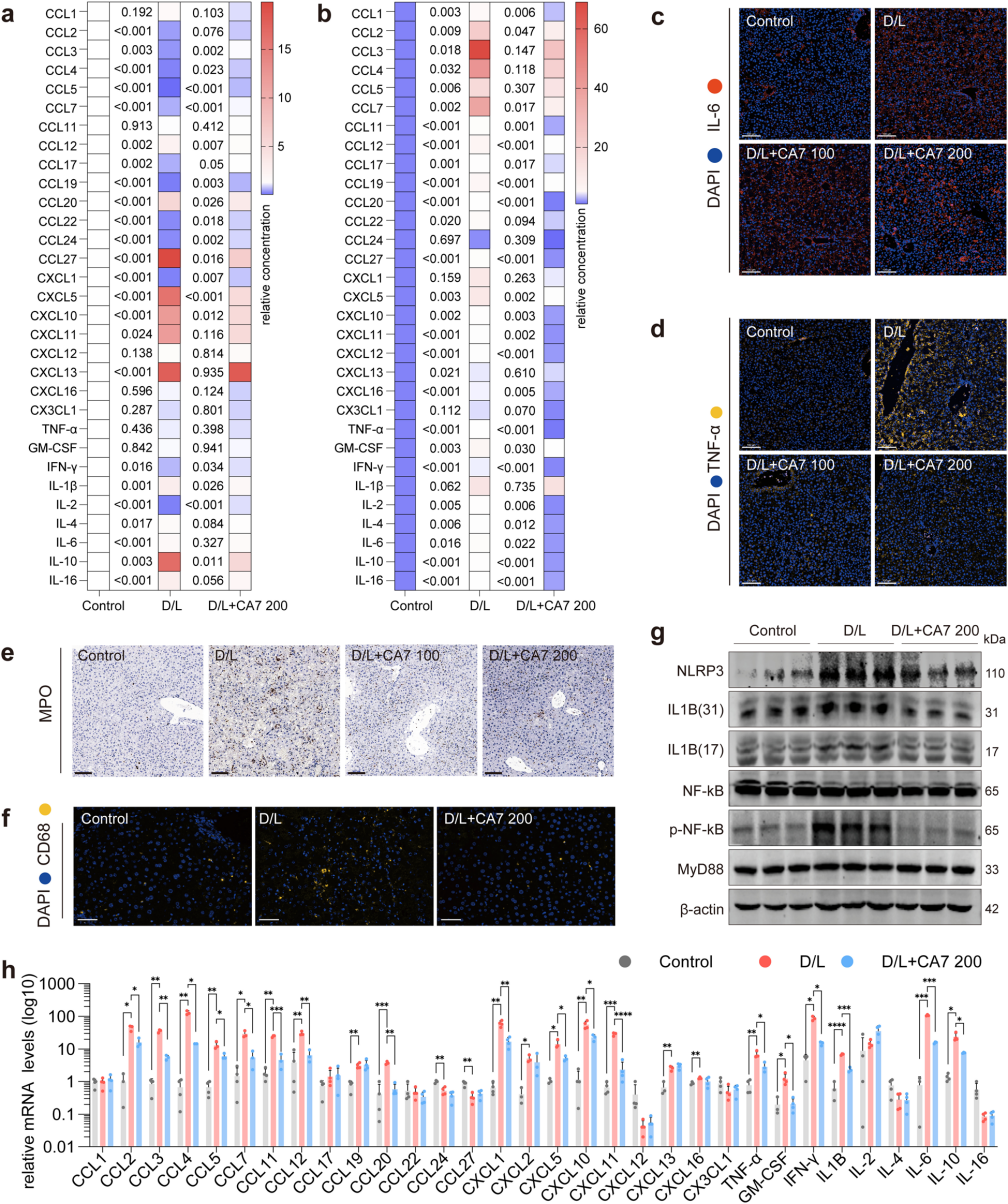
CA7 suppresses cytokine storm and inflammation for hepatoprotection. **a**–**b** Quantification of mice serum a or liver b for 31 key chemokines. All cytokine levels in mice were normalized to the average level of the control group mice. Unpaired t-test was used to assess statistical significance between the two groups. *n* = 3–4 samples/group. **c**-**d** Representative images of immunofluorescence staining for TNF-α c and IL-6 d in mouse liver tissues. Scale bar: 100 μm. Nuclei (blue, DAPI). **e** Representative images of IHC staining for MPO in mouse liver tissues. Scale bar: 100 μm. **f** Representative images of immunofluorescence staining for F4/80 in mouse liver tissues. Scale bar: 100 μm. Nuclei (blue, DAPI). **g** Western blot analysis of protein expression levels of NLRP3, IL-1β, NF-κB, p-NF-κB and MyD88 in mouse liver tissues. β-actin was used as control. *n* = 3 samples/group. **h** qPCR analysis of mRNA expression levels of key cytokines in mouse liver tissues. β-actin was used as control. *n* = 3–4 samples/group. Mean ± SD. One-way ANOVA

As shown in Fig. 4a and b, D/L–challenged mice exhibited a pronounced cytokine storm in the liver, a hallmark of ALF that propagates systemic inflammation and culminates in multi-organ failure [34]. Specifically, all 31 cytokines and chemokines analyzed were significantly dysregulated in both serum and liver tissues of ALF mice compared with controls (Fig. 4a, b). Notably, CA7 treatment restored most of these chemokine levels to near those observed in control animals, specific ones— including Ccl3, Ccl4, Ccl5, and Ccl7—were markedly reduced. This decrease may reflect the recruitment of peripheral monocytes/neutrophils into the injured liver, leading to their depletion from the circulation and suggesting a state of peripheral immunosuppression. In contrast, CA7 treatment reversed this trend, and no such immunosuppressive signature was observed in the serum (Fig. 4a). Consistent with these findings, CA7 significantly reduced the levels of multiple cytokines in liver tissue compared to the model group, in line with its protective effect against hepatic inflammation and injury (Fig. 4b).

Immunofluorescence staining further confirmed robust accumulation of the proinflammatory cytokines TNF-α and IL-6 in ALF livers, both of which were markedly suppressed following CA7 treatment (Fig. 4c, d). In parallel, IHC analysis of myeloperoxidase (MPO) revealed a pronounced infiltration of MPO-positive cells in ALF mice, an effect that was sharply attenuated by CA7 administration (Fig. 4e). Consistently, immunofluorescence staining further showed that CA7 reduced the D/L-induced expansion of hepatic macrophages, highlighting its suppressive effect on innate immune cell accumulation (Fig. 4f). These anti-inflammatory effects were further supported at the transcriptional level, as CA7 significantly downregulated the mRNA expression of multiple inflammatory cytokines compared with the ALF group (Fig. 4h).

Since nuclear factor kappa-B (NF-κB) activation is a pivotal driver of inflammatory cytokine production and NOD-like receptor thermal protein domain associated protein 3 (NLRP3) inflammasome activation—leading to IL-1β maturation and propagation of the cytokine storm [35], we next examined this signaling axis. As shown in our data, NF-κB signaling and downstream NLRP3/IL-1β activation were markedly induced in ALF mice, whereas CA7 treatment effectively suppressed activation of this inflammatory cascade (Fig. 4g).

Collectively, these results indicate that CA7 alleviates ALF primarily by reshaping the inflammatory liver microenvironment, thereby suppressing cytokine storm and inflammatory cell infiltration, rather than acting solely through direct hepatocyte-protective mechanisms.

### Neutrophil transcriptional reprogramming underlies CA7-mediated suppression of oxidative stress and inflammation

In ALF, the pathological overproduction of ROS acts as a central executor of oxidative stress, directly contributing to mitochondrial dysfunction, cytokine storm and propagation of inflammatory injury. To identify the cellular sources of ROS targeted by CA7, we first evaluated its effects in H₂O₂-stimulated HepG2 hepatocytes and phorbol 12-myristate 13-acetate (PMA)-activated RAW264.7 macrophage models. Although CA7 reduced ROS levels in both cell types (Fig. S3a, e), a critical functional distinction was observed. In hepatocytes, CA7 failed to prevent H₂O₂-induced cytotoxicity, in contrast to the direct antioxidant N-acetylcysteine (NAC) (Fig. S3b–d). By contrast, in macrophages, CA7 not only suppressed ROS accumulation but also potently inhibited the induction of key inflammatory mediators, including TNF-α, IFN-γ, and Ccl4 (Fig. S3f). These results suggest that the hepatoprotective effects of CA7 in ALF mice are unlikely to result from direct cyto-protection of hepatocytes, but instead arise from modulation of immune cells-derived oxidative and inflammatory responses. This cell-type-specific action aligns with our earlier finding that CA7 acts by orchestrating a suppressive program in the liver microenvironment, collectively inhibiting cytokine storm and inflammatory responses.

**Fig. 5:**
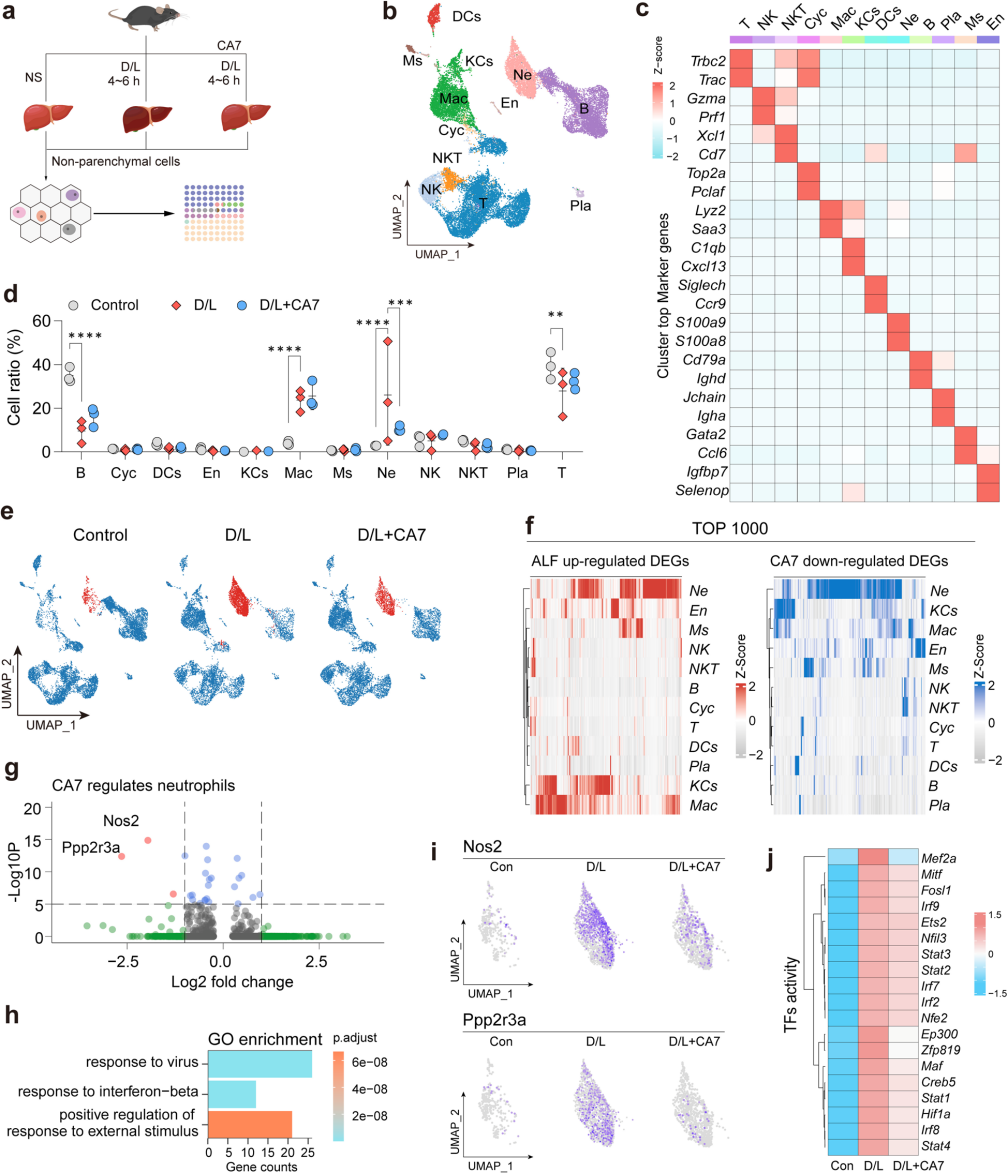
Neutrophil transcriptional reprogramming underlies the therapeutic effect of CA7 in ALF. **a** Schematic overview of the experimental design of scRNA-seq analysis of liver NPCs from healthy control, D/L-induced ALF, and CA7-treated ALF mice (*n* = 3 samples/group). **b** UMAP visualization showing unsupervised clustering of liver NPCs based on single-cell transcriptomes. **c** Heatmap of canonical marker gene expression used for cell-type annotation, with expression values scaled and centered across cells. **d** Bar graph summarizing the relative proportions of major immune and non-immune cell populations across experimental groups. Mean ± SD. One-way ANOVA. **e** UMAP projections colored by experimental group, highlighting neutrophils (red) and all other cell types (blue), illustrating changes in neutrophil abundance following D/L challenge and CA7 treatment. **f** Clustered heat map plotting the average expression of the 1000 most significantly up-regulated DEGs in each cell type in the ALF mice compared with control mice. **g** Volcano plot displaying differentially expressed genes in neutrophils between CA7-treated and ALF mice, highlighting key downregulated inflammatory and oxidative stress–related genes. **h** GO enrichment analysis of downregulated genes in neutrophils. **i** Feature plot showing Nos2 and Ppp2r3a expression in neutrophils across different groups. **j** TFs activity analysis in neutrophils

To further elucidate the immune cell populations underlying CA7-mediated hepatoprotection in vivo, we performed single-cell RNA sequencing (scRNA-seq) on liver immune cells isolated from healthy controls, ALF mice, and CA7-treated ALF mice (Fig. 5a). Based on established marker gene expression, we identified 12 major cell populations, including T cells (*Trac*), NK cells (*Prf1*), NKT cells (*Xcl1*), proliferating cells (*Top2a*), macrophages (*Lyz2*), Kupffer cells (*Cxcl13*), dendritic cells (*Siglech*), neutrophils (*S100a8*), B cells (*Cd79a*), plasma cells (*Jchain*), mast cells (*Gata2*), and endothelial cells (*Selenop*) (Fig. 5b, c). Comparative analysis revealed a marked expansion of myeloid cell populations—particularly macrophages and neutrophils, accompanied by a significant reduction in lymphocyte subsets, such as B cells and T cells in ALF mice. Notably, CA7 treatment effectively attenuated the ALF-induced expansion of neutrophils (Fig. 5d, e).

Differential expression gene (DEG) analysis between ALF and control groups identified the top 1000 upregulated DEGs, which were predominantly expressed in macrophages and neutrophils, indicating that these populations undergo the most profound transcriptional changes during ALF (Fig. 5f). Importantly, comparison between CA7-treated and ALF model groups revealed that neutrophils exhibited the greatest number of downregulated differentially expressed genes following CA7 treatment, identifying them as a preferential cellular target of CA7 (Fig. 5f). To further characterize CA7-induced transcriptional changes in neutrophils, we directly compared the DEGs between the CA7-treated and ALF mice. Among all DEGs, Nos2 (encoding inducible nitric oxide synthase, iNOS) emerged as the most significantly downregulated gene following CA7 treatment (Fig. 5g, i).

Nos2 is a key regulator of inflammatory mediator production and oxidative stress, and its overactivation drives liver injury by promoting excessive nitric oxide and ROS generation [36, 37]. The second most significantly downregulated gene was *Ppp2r3a* (Fig. 5g, i), which has been associated with necroptosis, post-inflammatory fibrosis, stress and proliferation [38–40]. Gene Ontology (GO) enrichment analysis further demonstrated that CA7 significantly suppressed neutrophil activation, with notable downregulation of inflammation-related pathways and interferon response signatures (Fig. 5h). Finally, transcription factor (TF) activity analysis revealed that CA7 inhibited key TFs, including members of the STAT and IRF family as well as HIF1α, all of which are critically involved in neutrophil activation and oxidative inflammatory responses [41, 42] (Fig. 5j).

In summary, scRNA-seq analysis revealed that neutrophils exhibit the most pronounced transcriptional and functional responses to CA7 in ALF. CA7 suppresses neutrophil activation by coordinately downregulating oxidative stress–associated and pro-inflammatory gene programs.

### CA7 alleviates ALF by suppressing ROS, thereby inhibiting NETosis

Given the established role of NETosis, a process by which activated neutrophils release NETs, in the pathogenesis of ALF [11–13], we investigated whether CA7 modulates this neutrophil effector mechanism. Human peripheral blood neutrophils were isolated, pretreated with CA7, and then stimulated with PMA to induce NETosis.

**Fig. 6:**
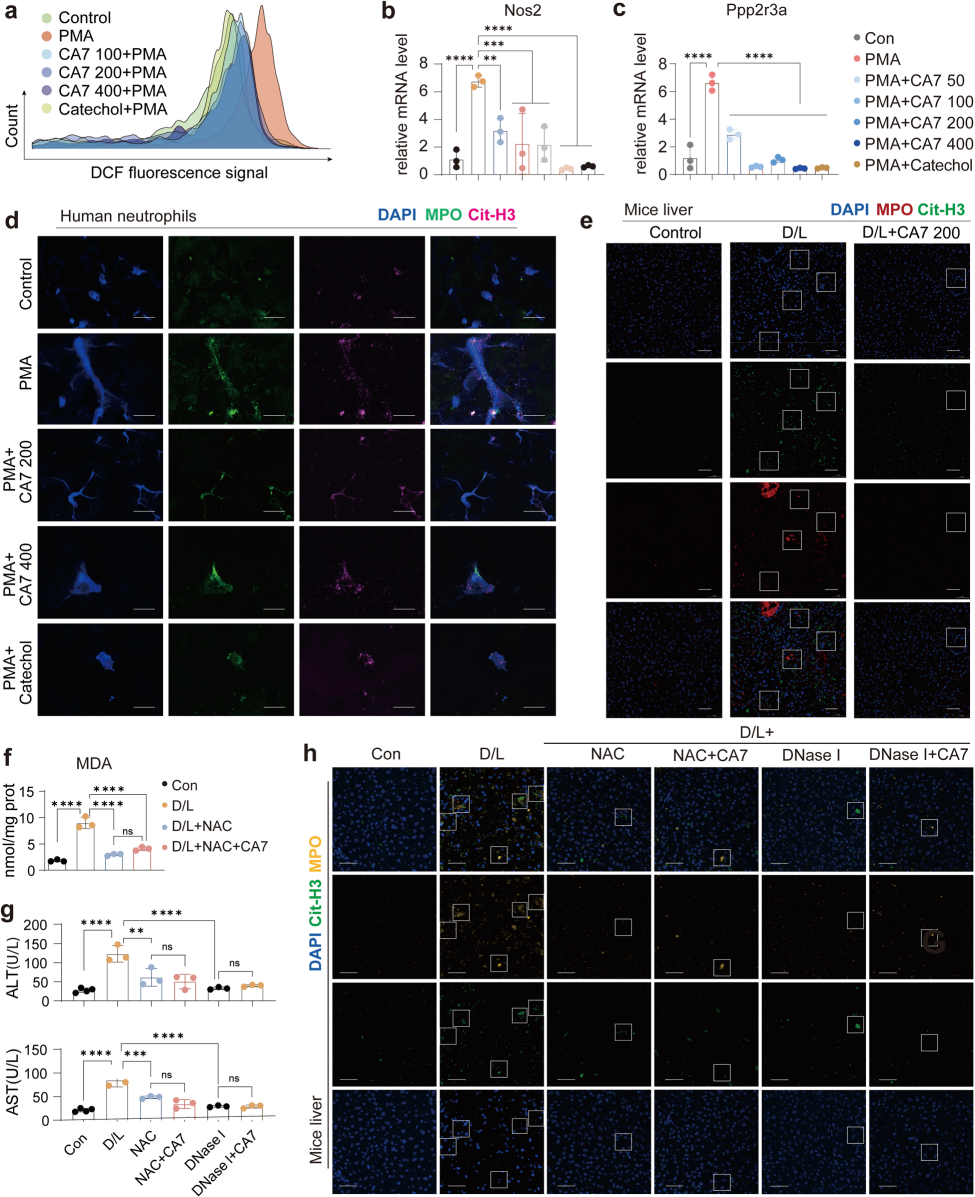
CA7 alleviates ALF by suppressing ROS and subsequent NETosis. **a** After human neutrophils were stimulated with PMA for 3 h, flow cytometry was used to detect neutrophil ROS levels. Similar results were obtained from three independent experiments. **b** qPCR analysis of the effect of CA7 on Nos2 mRNA levels in an in vitro NETs formation model. The significance of each group and the PMA-treated group is shown. *n* = 3. Mean ± SD. One-way ANOVA. **c** qPCR analysis of the effect of CA7 on Ppp2r3a mRNA levels in an in vitro NETs formation model. The significance of each group and the PMA-treated group is shown. β-actin was used as control. **d** Representative images of NETosis staining in human neutrophils, stained with DAPI for nuclei (blue). Scale bar: 50 μm. **e** Representative images of MPO and CitH3 staining in mouse liver sections, stained with DAPI for nuclei. Scale bar: 50 μm. **f** Levels of MDA in mouse liver homogenates (*n* = 3 samples/group). Mean ± SD. One-way ANOVA. **g** Scatter plot of serum ALT and AST levels (*n* = 3 samples/group). Mean ± SD. One-way ANOVA. **h** Representative images of MPO and CitH3 staining in mouse liver sections, stained with DAPI for nuclei. Scale bar: 50 μm

We first asked whether CA7 could attenuate the ROS burst associated with PMA-induced NETosis. Flow cytometric analysis revealed a sharp increase in intracellular ROS shortly after PMA stimulation, which was strongly suppressed by CA7 pretreatment (Fig. 6a). Moreover, in this classical NETosis model, we observed marked upregulation of Nos2 and Ppp2r3a—genes previously linked to neutrophil activation in our scRNA-seq data—and CA7 pretreatment potently inhibited their expression (Fig. 6b, c). To directly visualize NETosis, immunofluorescence co-staining for citrullinated histone H3 (CitH3) and MPO was performed [43]. PMA stimulation robustly induced CitH3/MPO co-localization, indicative of NET release, whereas CA7 substantially reduced this signal (Fig. 6d). A similar inhibitory effect of CA7 on NETosis was observed in mouse liver sections following D/L-induced ALF (Fig. 6e). Quantitative assessment using Sytox Green–based extracellular DNA measurement confirmed that PMA triggered significant NET release, which was effectively suppressed by CA7 (Fig. S4).

Collectively, these results demonstrate that CA7 effectively restrains NETosis both in vitro and in vivo by attenuating early ROS accumulation.

To determine whether the hepatoprotective effect of CA7 depends on inhibition of ROS and NETs, we performed interventional studies using the ROS scavenger NAC and NETs scavenger Deoxyribonuclease I (DNase I). ALF mice were treated with NAC alone or in combination with CA7, and hepatic MDA levels and serum ALT/AST were measured. NAC monotherapy significantly reduced MDA and ALT/AST compared with the D/L model, whereas co-treatment with CA7 and NAC did not provide additional improvement (Fig. 6f, g). Separately, DNase I was used to degrade NETs, and IMMUNOFLUORESCENCE analysis confirmed that co-treatment with CA7 and DNase I did not further suppress NETosis compared with CA7 alone (Fig. 6 h). Together, these results indicate that the hepatoprotective effect of CA7 is primarily mediated through suppression of ROS and the subsequent inhibition of NET formation.

In summary, ROS produced by hepatic cells activate neutrophils to undergo NETosis, which amplifies oxidative stress and cytokine release, exacerbating liver injury in ALF. CA7 interrupts this cycle by reducing ROS accumulation and suppressing NET formation, thereby mitigating oxidative stress, inflammation, and liver damage.

## Discussion

ALF is a life-threatening condition characterized by excessive oxidative stress and dysregulated innate immunity, resulting in hepatocyte injury and systemic inflammation [1, 44]. In this study, we identify CA7, a cinnamic acid derivative, as a novel therapeutic agent capable of mitigating ALF. While cinnamic acid and its derivatives have long been used in pharmaceuticals, cosmetics, and food science for their antioxidant properties [18–20, 45, 46], their potential as anti-ALF therapeutics has been largely unexplored. Here, we demonstrate that CA7 effectively reduces liver injury and improves survival in a murine ALF model, providing a clear pharmacological advantage by simultaneously alleviating oxidative stress and modulating inflammatory responses. These findings highlight CA7 as a promising candidate for further preclinical development in the treatment of ALF.

Neutrophils are key mediators of innate immunity and play a critical role in the pathogenesis of ALF, contributing to hepatocyte injury and inflammatory amplification [8–10]. Recent studies suggest that NETosis contributes to ALF progression, but most investigations have focused on mechanistic insights and remain largely at the basic research level [47–49]. Advances in single-cell sequencing technologies now allow more precise characterization of immune cell responses in ALF [50, 51]. Leveraging these tools, we employed scRNA-seq and functional assays to systematically evaluate CA7's cellular targets, confirming its action on neutrophils and its ability to modulate immune-mediated liver injury.

**Fig. 7:**
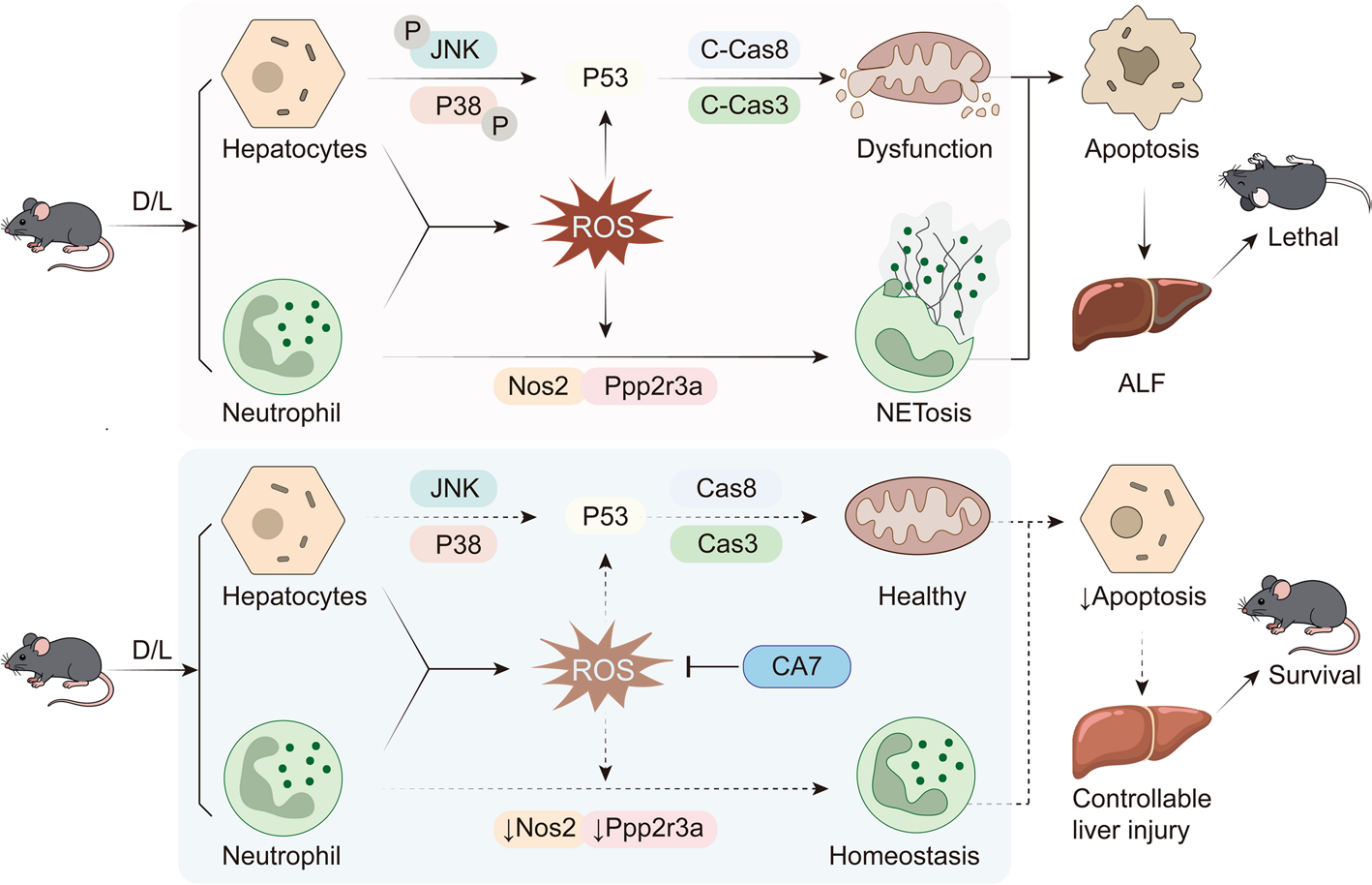
Schematic illustration of CA7 protecting against ALF by inhibiting ROS and subsequent NETosis

Beyond its therapeutic potential, our study also provides mechanistic insights into ALF pathogenesis and CA7's mode of action. We show that ROS triggers NET formation, which amplifies oxidative stress and inflammatory cytokine release, forming a self-perpetuating injury loop. CA7 interrupts this cycle by attenuating ROS accumulation and downregulating NETosis-related genes, such as Nos2 and Ppp2r3a, thereby protecting hepatocytes in vivo. scRNA-seq analysis further revealed that neutrophils exhibit the most pronounced transcriptional and functional responses to CA7, underscoring the relevance of targeting these immune cells to achieve therapeutic efficacy. These mechanistic findings provide a foundation for understanding how CA7 exerts its dual antioxidant and immunomodulatory effects (Fig. 7).

Despite these promising results, several limitations should be noted. First, while our murine ALF model recapitulates key pathological features, it cannot fully reflect human disease, and species differences in neutrophil biology and drug metabolism require further validation. Second, the optimal therapeutic window for CA7 administration remains to be defined, including potential efficacy post-onset. Third, the precise molecular target of CA7 remains unidentified. Its inhibition of ROS and NETosis appears NRF2-independent and may involve NADPH oxidase, mitochondrial ROS, or other redox-regulatory pathways. Addressing these gaps will be essential for translating CA7 into clinical application.

In conclusion, this study positions CA7 as a pioneering therapeutic candidate bridging antioxidant and immunomodulatory strategies in ALF. By disrupting the ROS-NETosis axis and mitigating hepatocyte injury, CA7 not only offers mechanistic insights into ALF pathogenesis but also provides a foundation for future pharmacological development. Our findings underscore the potential of CA derivatives as clinically relevant interventions and highlight directions for further mechanistic and translational research in liver disease.

## Materials and methods

### Animals and treatment

Male C57BL/6 mice (8 weeks old, 20–22 g) were sourced from Vital River Laboratory Animal Technology Co. Ltd (SCXK2021-0006, Beijing, China) and allowed to adapt to laboratory conditions for 7 days in a controlled environment (12 h light/dark cycle, 24 °C ± 1 °C). All experimental procedures were conducted in accordance with the guidelines of the Institutional Animal Care and Use Committee ((IACUC) at Huazhong University of Science and Technology (Approval No. [2023] IACUC Number: 4411).

Mice were randomly allocated into seven groups: Control, Negative Control, Model, CA7 (50, 100, or 200 mg/kg), along with a Silymarin group (200 mg/kg, Sigma, S0292). CA7 and silymarin were suspended in 0.5% CMC-Na and administered orally for 10 days. One hour after the final dose, ALF was induced via intraperitoneal injection of D-GalN (500 mg/kg) and LPS (5 μg/kg, Sigma, G1639/L2630) in all groups except controls, which received PBS. Mice were euthanized 4.5 h later for liver and blood collection; body and liver weights were recorded, and liver images captured.

For survival studies, an additional group received a single intraperitoneal injection of CA7 (in PBS with 10% DMSO) 1 h before D/L challenge. Mice were monitored continuously, and those showing severe lethargy or convulsions were humanely euthanized, with the time of euthanasia recorded as the time of death.

### Mouse chemokine detection

A multiplex bead-based immunoassay (Bio-Plex Pro Mouse Chemokine 31-Plex Kit, Bio-Rad, 1,209,159) was used to simultaneously detect multiple chemokines as per the vendor's protocol. Liver-derived chemokine levels were normalized to the total protein content.

### ROS staining in mouse liver tissue

Following sacrifice, liver tissues were rapidly excised and snap-frozen in liquid nitrogen. Cryosections were incubated with ROS-specific staining reagent at 37 °C for 35 min in the dark, washed three times with PBS (5 min each), and then stained with DAPI for 10 min at RT. Cryosections were washed again, excess liquid removed, mounted with anti-fade medium, and imaged using an Olympus CKX53 microscope.

### Preparation of liver cell suspension

Mouse livers were minced with scissors, transferred to a lysis tube, and digested with 10 mL of enzyme solution containing Collagenase IV (0.4 mg/mL, Cat: 091GR001), Protease E (0.1 mg/mL, Cat: HY-114158), and DNase I (0.01 mg/mL, Cat: 10,104,159,001). Following mechanical disruption using a Miltenyi dissociator, the digest was subjected to sequential centrifugation at 50 g for 4 min and 600 g for 8 min at 4 °C to isolate viable cells. The resulting pellet was treated with cold Ammonium-Chloride-Potassium (ACK) lysis buffer, filtered through a 40 μm mesh, and enriched for non-parenchymal cells via density centrifugation with fragment removal solution (Miltenyi, 130–109–398). After a final wash step (1000 g, 10 min), cells were resuspended in complete culture medium and depleted of dead cells using the Dead Cell Removal Kit (Miltenyi, 130–090–101).

### scRNA-seq analysis

Library preparation and sequencing: Single-cell suspensions were barcoded using the Single Cell Sample Multiplexing Kit (BD Biosciences, 633,793) and captured using BD Rhapsody Express and BD Rhapsody Cartridge chips (633,731 & 633,733). Whole Transcriptome Amplification libraries and Sample Tag Index PCR products were generated following the manufacturer’s protocol (BD Biosciences, 633,773 & 633,801; Beckman Life Sciences, A63880). The WTA index and sample tag index PCR libraries were sequenced on the MGISEQ-2000 platform for paired-end reads, generating raw sequencing data [52].

Read alignment: Bioinformatic processing began with alignment of FASTQ files to the mm 10 reference genome (reldata-cellranger-mm10-3.0.0, sourced from 10X Genomics) using Cell Ranger v5.0.1. Feature and cell barcode matrices were generated using the BD Rhapsody module (1.9.1). Data were normalized, and downstream analyses, including cell quality filtering, clustering, differential expression, and dimensionality reduction, were conducted using Seurat v4.2 in R. For cell quality control, the following parameters were applied: 200 < nFeature_RNA < 6000, 500 < nCount_RNA < 40,000, log10GenesPerUMI > 0.80, percent.mt < 20% and percent.rp < 20%. Clustering was performed using the FindClusters function, followed by UMAP embedding via RunUMAP. Cluster-specific marker genes were identified using the FindMarkers function.

Functional annotation was carried out through GO enrichment analysis (adjusted *P* < 0.01, Bonferroni-corrected), implemented with the clusterProfiler package. The compareClusters and enrichGO functions were utilized to identify enriched GO terms. Differentially expressed genes between different groupings were also conducted [53].

TFs regulatory analysis: Gene regulatory networks were reconstructed from scRNA-seq data using SCENIC [54], a computational framework that reconstructs gene regulatory networks from single-cell RNA sequencing data. Further analysis focused on TFs whose activity was significantly upregulated.

### Software used for graphing and visualization

Graphs and visualizations were created using the R packages ggplot2 (3.3.6) for general plotting; EnhancedVolcano (1.14.0) for volcano plots. GraphPad Prism 8 software was also utilized for additional plotting. Vector graphics were created using Adobe Illustrator software. Some vector graphic elements were sourced from the Freepik website (https://www.freepik.com/).

### Neutrophil isolation and in vitro stimulation protocol

Neutrophils were purified from anticoagulated peripheral blood (JiningBio, JN-CC6058) through density gradient centrifugation. Briefly, fresh anticoagulated peripheral blood, saline and erythrocyte sedimentation solution were mixed in a 1:1:1 ratio. After standing at room temperature (RT) for 30–40 min, red blood cells can be seen in the sediment at the bottom of the tube. The resulting supernatant, enriched in leukocytes, was collected and gently layered over neutrophil separation medium (NSM) in 15 mL conical tubes to establish a distinct interface. Following centrifugation at 800 g for 30 min at RT, two discrete bands appeared: the upper band consisted of mononuclear cells, and the lower band contained polymorphonuclear neutrophils. The neutrophil-rich layer was harvested and transferred to fresh tubes, washed with PBS, and centrifuged to remove residual debris. The pellet was resuspended in lysis buffer to eliminate contaminating red blood cells (RBCs), followed by multiple rounds of washing and centrifugation. The final pellet comprised highly purified neutrophils, ready for downstream stimulation assays.

### Flow cytometry detection of ROS levels in neutrophils

The total ROS levels were evaluated with probe DCFH-DA (Beyotime, S0035S). After treatment with CA7 alone for 30 min, then PMA (50 nM, Beyotime) were added to plate for 4 h. At last, the neutrophils were stained with DCFH-DA (10 μM, Beyotime) for 30 min in the dark. Flow cytometric analysis with DCFH-DA staining was performed to assess the level of ROS in the neutrophils.

### NETs staining protocol for cells and tissues

For immunofluorescent detection of NETs, pretreated neutrophils (1.5 × 10⁶ cells/well) were plated onto glass coverslips in 24-well plates. Cells were washed with PBS, fixed in 4% paraformaldehyde for 15 min at room temperature, permeabilized with 0.2% Triton X-100 (Sigma, V900502) for 10 min at 37 °C, and blocked with 3% bovine serum albumin (BSA) for 30 min at 37°C. Primary antibodies against citrullinated histone H3 (CitH3; 1:400, Proteintech, 13,754–1-AP) and myeloperoxidase (MPO; 1:200, Proteintech, 66,177-1g) were applied and incubated at room temperature for 2 h. After washing, cells were incubated with fluorophore-conjugated secondary antibodies (Cy3/Cy5 or FITC/Alexa Fluor 488; 1:200 to 1:500, Abcam, ab6939, ab150077, or ab97035) for 50 min at room temperature. Nuclear DNA was counterstained with Hoechst 33,342 (Beyotime, 33,342) for 10 min at 37°C. Fluorescent signals were visualized and captured using a Nikon AX/AX R confocal microscope system. Immunofluorescence staining of formalin-fixed, paraffin-embedded tissue sections followed an identical protocol, with appropriate antigen retrieval and blocking steps.

### Statistical analysis

Data are presented as the mean ± standard deviation (SD). Statistical evaluations were conducted using GraphPad Prism version 9.0, and differences were deemed statistically significant at P < 0.05.

### Compound source

The synthesis of CA7 and other methodological details are provided in the Supplementary Information.

## Supplementary Information

Supplementary Material 1.

## Data Availability

The dataset generated during the current study have been deposited in the China National Center for Bioinformation, under accession number PRJCA060138.
