# Supplementary material for: Amelioration of acute liver failure by a cinnamic acid derivative through inhibition of the ROS–NETosis axis
Source: Mol Biomed. 2026 Apr 13;7:50. doi: 10.1186/s43556-026-00448-x (PMC13076717; doi:10.1186/s43556-026-00448-x)
Supplement: Supplementary file 1 — Supplementary Material 1. [file 43556_2026_448_MOESM1_ESM.docx]

**Amelioration of acute liver failure by a cinnamic acid derivative** **through inhibition of the ROS–NETosis axis**

Jie Yin^a#^, Longjie Ding^a#^, Ziming Zhao^a#^, Xia Chen^b^, Jianzheng Huang^a^, Yang Xiao^a^, Lianghu Gu^a^, Xiaotian Zhang^a^*, Qingyi Tong^a^*, Yonghui Zhang^a^*.

**Authors’ Affiliations:**

1. Hubei Key Laboratory of Natural Medicinal Chemistry and Resource Evaluation, School of Pharmacy, Tongji Medical College, Huazhong University of Science and Technology, Wuhan, Hubei, China.
2. Institute of Pharmaceutical Process, Hubei Province Key Laboratory of Occupational Hazard Identification and Control, School of Medicine, Wuhan University of Science and Technology, Wuhan 430065, China.

* **Corresponding author**: Yonghui Zhang, Qingyi Tong and Xiaotian Zhang;

Tel.: +86-27-8369286; Fax: +86-27-83692762.

E−mail: zhangyh@mails.tjmu.edu.cn (Y. Zhang), qytong@hust.edu.cn (Q. Tong) and [zhangxt997@126.com](mailto:zhangxt997@126.com) (X. Zhang)

# These authors made equal contributions to this work.

Supporting Information

1. Materials and Methods

1.1 H&E staining and histological scoring

The liver tissues were fixed in 4% paraformaldehyde and embedded in paraffin. Later, the fixed liver tissues were cut into 5-μm-thick sections. These sections were stained with H&E to evaluate the liver histopathological injury. For histological Scoring, three random fields of view were captured from each H&E-stained section and scored based on the severity of hepatic hemorrhage, hepatocyte death, inflammatory cell infiltration, and disruption of liver architecture.

The scoring criteria were as follows: 1 point (extreme deterioration, lethal ALF), 2 points (severe deterioration, ALF), 3 points (severe liver damage), 4 points (liver damage) and 5 points (normal). This assessment was independently conducted by three researchers with extensive expertise in liver injury studies. To minimize subjective bias during the evaluation process, researchers remained blinded to the specific group assignments of the samples.

1.2 Measurement of ALT, AST, MDA and SOD levels

The serum levels of ALT and AST in mice were measured by assay kits (Nanjing Jiancheng, C009-2-1&C010-2-1). The liver tissues of mice were homogenized and the supernatant were used to detect the levels of MDA and SOD using assay kits (Nanjing Jiancheng, A003-1-2& A001-3-2). All operations were performed according to the manufacturer's instructions.

1.3 TUNEL staining

TUNEL staining was performed using an In Situ Cell Death Detection Kit (Roche, 11684817910) according to the manufacturer's instructions. Specifically, paraffin sections were pre-treated according to the above H&E staining method, and then treated with proteinase K working solution at 37℃ for 25 min to repair the antigen and subsequently washed with PBS. Next, the membrane-permeabilizing solution was used to cover the tissue membrane, which were incubated at RT for 20 min, followed by another PBS wash. Then, TDT and dUTP were mixed in the ratio of 1:9, covered the tissue and incubated at 37℃ for 2 h. Finally, DAPI was used to stain the nucleus. Nikon AX Confocal Microscope System was used for imaging.

1.4 Immunohistochemistry

For IHC, sections were blocked with 3% BSA or 10% rabbit serum in PBS for 30 min at RT, then incubated overnight at 4℃ with the following primary antibodies: MPO (1:2000, Abcam, ab208670), CC3 (Asp175) (1:400, CST, 9661). On the second day, the section was incubated with HRP-labeled secondary antibody for 50 min at RT, followed by the addition of freshly prepared DAB chromogenic solution. The nuclei were stained with hematoxylin, and the sections were dehydrated and mounted with neutral gum. Imaging was conducted using an Olympus CKX53 microscope.

1.5 Immunofluorescence histochemistry

Paraffin sections were pretreated and blocked according to the above immunohistochemical method. After the preparation, sections were incubated overnight at 4℃ with the following primary antibodies: LC3A/B (1:500, CST, 12741), IL-6 (1:200, Absin, abs135607) and TNF-α (1:500, CST, 11948), and in the next day incubated with TRITC-labeled secondary antibody (1:2000, Thermofisher, PA1-28565 & T2769) for 50 min at RT. Lastly, the nuclei were stained with DAPI staining solution (Solarbio, C0065) at RT for 10 min, followed with the blockage of the sections using anti-fluorescence quenching blocking reagent (Solarbio, S2110). Finally, Nikon AX/AX R Confocal Microscope System was used for imaging.

1.6 Cell culture

HepG2 and RAW 264.7 cell lines was obtained from Procell. HepG2 cells were maintained in MEM medium (Procell, PM150410) supplemented with 10% fetal bovine serum (FBS). RAW 264.7 cells were maintained in high-glucose Dulbecco's modified Eagle medium (DMEM, Procell, PM150210), supplemented with 10% fetal bovine serum (FBS). The neutrophils separated from human peripheral blood were maintained in 1640 medium (Procell, Cat:150110) without fetal bovine serum (FBS). All cell lines were cultured in a 37℃ incubator with 5% CO_2_. Cell culturing, cryopreservation, and thawing were all meticulously conducted in strict accordance with standard operating procedures. Short tandem repeat profiling and contamination assessment of the cell lines were performed every six months. The results verified the authenticity of the cell lines and confirmed their contamination-free status.

1.7 Cellular model of oxidative stress and inflammation

To stimulate the HepG2 cell, the cells were pre-treated with either 100 μM, 200 μM or 400 μM CA7 for 30 min, followed by the addition of 500 μM hydrogen peroxide (H_2_O_2_). After 6 h, the levels of ROS were measured.

To stimulate the RAW 264.7 cells, the cells were pre-treated with either 100 μM or 200 μM CA7 for 30 min, followed by the addition of 50 nM PMA. After 4 h, the levels of ROS were measured.

To detect ROS, DCFH-DA was diluted to 10 μM in serum-free medium. Lately, we removed the culture medium, and incubated the cells with the DCFH-DA staining solution at 37℃ for 30 min. After the incubation, the cells were washed three times with serum-free medium. The DCF fluorescence signal were collected using an Olympus CKX53 microscope and a flow cytometry with an excitation wavelength of 488 nm and an emission wavelength of 525 nm.

1.8 Cell Viability assay

Cell viability was measured with a Cell Counting Kit-8 (CCK-8) (Target Mol, C0005). Briefly, cells were seeded in 96-well plates and incubated with or without drugs. After 24 h, 10% (v/v) CCK-8 regent was added and incubated in dark at incubator for 3 h. The optical density values were measured at 450 nm.

**1.9 Transmission electron microscope (TEM)**

Samples of mouse liver tissue, approximately 1–2 mm³ in size, were placed in a 2.5% glutaraldehyde solution and stored at 4℃. Each fixed tissue sample was then rinsed three times with 0.1 M phosphate buffer (pH = 7.4) for 15 min each. Subsequently, the samples were fixed with 1% osmic tetroxide in 0.1 M phosphate buffer (pH = 7.4) at room temperature (RT) for 2 h, followed by rinsing with 0.1 M phosphate buffer (pH = 7.4) three times, 15 min each time. Dehydration was achieved through a graded ethanol series (30%, 50%, 70%, 80%, 85%, 90%, and 100%, twice) for 15 min at each concentration. The samples were then infiltrated with increasing concentrations of epoxy resin in acetone (2:1, 1:1, and pure epoxy resin) at 37℃ for 12 h at each step. After infiltration, the samples were embedded in epoxy resin and polymerized at 60℃ for 48 h. Ultra-thin sections (100 nm) were cut using an ultramicrotome (Leica, Model UC7). Sections were then subjected to uranium-lead double staining by immersing them in a 2% saturated uranyl acetate aqueous solution for 15 min at RT followed by lead citrate staining for another 15 min at RT. Finally, the sections were dried overnight at RT and observed under transmission electron microscope (FEI, TECNAI G 20 TWIN).

1.10 RNA isolation and quantitative PCR (qPCR)

Total RNA from mouse livers or neutrophils were isolated using TRIzol Reagent (Thermofisher, 15596026) and cDNA was synthesized by SuperMix for qPCR (Vazyme, R223-01). The SYBR Green qPCR Mix was purchased from Biosharp (BL698A). The sequence of primers used in the study are listed in Supplemental Digital Content.

1.11 Western blots

To prepare the samples, tissues or harvested cells were homogenized using an appropriate RIPA buffer (Beyotime, P0013B). The lysates were incubated at 4°C for 20 min and then centrifuged at 12,000 g for 10 min at 4°C. Then the supernatant was collected, and the protein concentration was determined using a BCA protein assay kit (Beyotime, P0012S), SDS-PAGE protein loading buffer was added to the protein samples, which were then denatured at 95℃ for 10 min and stored at -20°C for further experiments. 10–30 μg of protein was separated by SDS-polyacrylamide gel electrophoresis and transferred onto NC membrane (Pall Corporation, 66485) in transfer buffer containing 20% methanol. The membrane was blocked with 5% nonfat dry milk (w/v) for 1–2 h, and subsequently incubated overnight at 4 ℃ with specific primary antibodies. After washing three times with PBST buffer solution, membranes were incubated with secondary antibody (1:15000) for 1 h. Lastly, the membranes were visualized using an imaging system (LI-COR, Odyssey CLx), and Image Studio analysis software was used for quantitative analysis of band strength.

The following antibodies were purchased from CST: CC3 (Asp175) (9661), Caspase-8 (9746), p53 (2524), Phospho-SAPK/JNK (Thr183/Tyr185) (4668), SAPK/JNK (9252), Phospho-p38 MAPK (4511), p38 MAPK (8690), Erk1/2 (9102), Phospho-Erk (Thr202/Tyr204) (9101), Phospho-mTOR (Ser2448) (2971), mTOR (2972), SQSTM1/p62 (39749), LC3B (2775), Beclin-1 (3495), Phospho-AMPKα (Thr172) (2535), AMPKα (5831), Phospho-SQSTM1/p62 (Ser349) (16177), KEAP1 (8047), NRF2 (12721), HO-1 (43966), NQO1 (62262), NLRP3 (15101), IL-1β (31202), NF-κB p65(8242), Phospho-NF-κB p65 (Ser536) (3033), MyD88 (4283), Anti-mouse IgG (H+L) (5257) and Anti-rabbit IgG (H+L) (5366). Antibodies from Santa Cruz included ATF4 (sc-390063), PGAM5 (sc-515880), ATP5B (sc-74549), and from Proteintech: PARP1 (66520-1-Ig), β-actin (66009-1-Ig), α-Tubulin (66031-1-Ig) and GAPDH (10494-1-AP).

Image Studio analysis software from the CLx Imaging System was used for quantitative analysis of Western blot band intensities.

1.12 Software used for graphing and visualization

Graphs and visualizations were created using the R packages ggplot2 (version 3.3.6) for general plotting; EnhancedVolcano (version 1.14.0) for volcano plots. GraphPad Prism 8 software was also utilized for additional plotting. Vector graphics were created using Adobe Illustrator software. Some vector graphic elements were sourced from the Freepik website (https://www.freepik.com/).

1.13 Fluorescence quantification of Sytox Green staining

Image-based quantification was performed using ImageJ software. For intensity measurements, multichannel immunofluorescence images were first split into individual color channels via Image, Color and Split Channels. The green channel was selected for analysis and converted to 8-bit grayscale to standardize pixel intensity values. Background fluorescence was minimized by applying a consistent threshold to highlight and select fluorescent regions corresponding to positive signals. To ensure objectivity, the same threshold settings were applied across all images within a given experiment. The following parameters were set for measurement: Area, Mean Gray Value, Standard Deviation, and Limit to Threshold. Fluorescence intensity was quantified by measuring the integrated density (IntDen = Area × Mean Gray Value) after background subtraction.

1. **Supplementary Figures and Legends**

**2.1 Synthesis of CA7**

To a stirred solution of commercially available cinnamic acid (15 mmol, 1.0 equiv.) in dry DCM (40 mL) was slowly added to oxalyl chloride (45 mmol, 3.0 equiv.) and DMF (0.75 mmol, 0.05 equiv.) dropwise at RT in a round bottom flask. The resulting mixture was stirred at RT for approximately 3 h and monitored by TLC analysis. After the reaction was completed, the solvent was removed by rotary evaporation. The concentration led to the corresponding cinnamoyl chloride, which were used directly for the next step without further purification.

To a stirred solution of methyl a-D-glucopyranoside (10 mmol, 1.0 equiv.) and dry pyridine (100 mmol, 10.0 equiv.) in dry DCM at -10 ℃ was added the above prepared cinnamoyl chloride (11 mmol, 1.1 equiv.). Then, the reaction mixture was allowed to warm to RT and stirred overnight. The reaction mixture was quenched with MeOH, and then the solvent was removed under vacuum. Thereafter, the mixture was acidified with 1 M HCl (aq.) and extracted with DCM approximately six times. The combined organic layer was dried over anhydrous Na_2_SO_4_, filtered and concentrated. The obtained crude products were purified by silica column chromatography (PE/EA = 3:1–1:2) to give esterification products CA7.

**
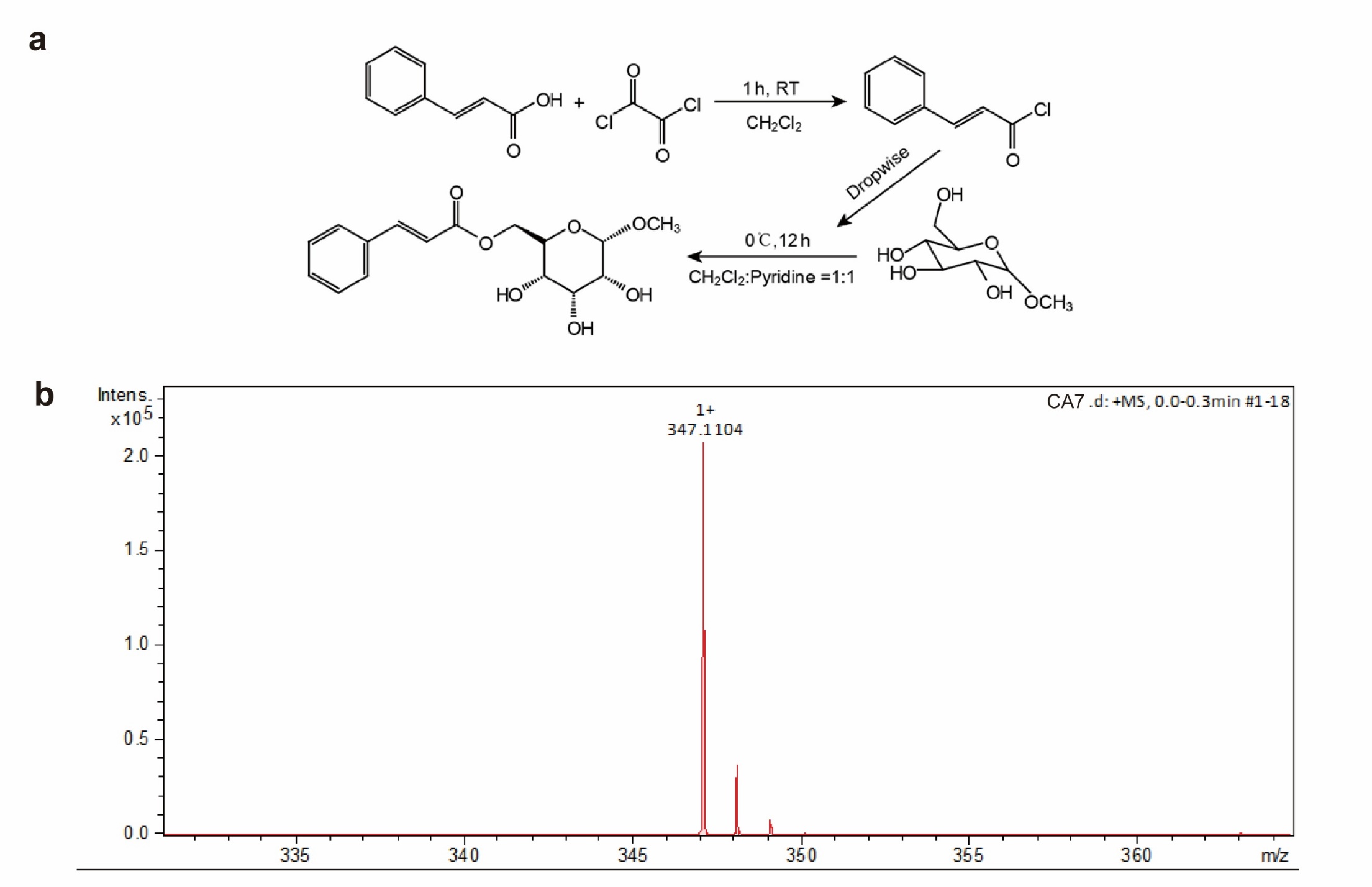
**

**Fig. S1** Schematic representation of the chemical synthesis route of CA7.

**a** Chemical synthesis route of CA7. **b** Mass spectrum of the purified CA7, confirming the expected molecular weight and compound identity.

**2.2 qPCR primers sequences**

| Gene | Forward primers | Reversed primers |
| --- | --- | --- |
| CCL1 | GCTTACGGTCTCCAATAGCTGC | GCTTTCTCTACCTTTGTTCAGCC |
| CCL2 | GCTACAAGAGGATCACCAGCAG | GTCTGGACCCATTCCTTCTTGG |
| CCL3 | ACTGCCTGCTGCTTCTCCTACA | ATGACACCTGGCTGGGAGCAAA |
| CCL4 | ACCCTCCCACTTCCTGCTGTTT | CTGTCTGCCTCTTTTGGTCAGG |
| CCL5 | CCTGCTGCTTTGCCTACCTCTC | ACACACTTGGCGGTTCCTTCGA |
| CCL7 | CAGAAGGATCACCAGTAGTCGG | ATAGCCTCCTCGACCCACTTCT |
| CCL11 | TCCATCCCAACTTCCTGCTGCT | CTCTTTGCCCAACCTGGTCTTG |
| CCL12 | GCTACAGGAGAATCACAAGCAGC | ACGTCTTATCCAAGTGGTTTATGG |
| CCL17 | CGAGAGTGCTGCCTGGATTACT | GGTCTGCACAGATGAGCTTGCC |
| CCL19 | TCGTGAAAGCCTTCCGCTACCT | CAGTCTTCGGATGATGCGATCC |
| CCL20 | GTGGGTTTCACAAGACAGATGGC | CCAGTTCTGCTTTGGATCAGCG |
| CCL22 | GTGGAAGACAGTATCTGCTGCC | AGGCTTGCGGCAGGATTTTGAG |
| CCL24 | ATTCCAGAAAACCGAGTGGTTAGC | GCATCCAGTTTTTGTATGTGCCTC |
| CCL27 | TTTCCTTGGCTGCGAATGTGGC | CTTGTTCCACGGATGCCAGCTT |
| CXCL1 | TCCAGAGCTTGAAGGTGTTGCC | AACCAAGGGAGCTTCAGGGTCA |
| CXCL2 | CATCCAGAGCTTGAGTGTGACG | GGCTTCAGGGTCAAGGCAAACT |
| CXCL5 | CCGCTGGCATTTCTGTTGCTGT | CAGGGATCACCTCCAAATTAGCG |
| CXCL10 | ATCATCCCTGCGAGCCTATCCT | GACCTTTTTTGGCTAAACGCTTTC |
| CXCL11 | CCGAGTAACGGCTGCGACAAAG | CCTGCATTATGAGGCGAGCTTG |
| CXCL12 | GGAGGATAGATGTGCTCTGGAAC | AGTGAGGATGGAGACCGTGGTG |
| CXCL13 | CATAGATCGGATTCAAGTTACGCC | GTAACCATTTGGCACGAGGATTC |
| CXCL16 | GCAGGGTACTTTGGATCACATCC | AGTTCACGGACCCACTGGTCTT |
| CX3CL1 | CAGTGGCTTTGCTCATCCGCTA | AGCCTGGTGATCCAGATGCTTC |
| TNF-α | GGTGCCTATGTCTCAGCCTCTT | GCCATAGAACTGATGAGAGGGAG |
| GM-CSF | AACCTCCTGGATGACATGCCTG | AAATTGCCCCGTAGACCCTGCT |
| IFN-γ | CAGCAACAGCAAGGCGAAAAAGG | TTTCCGCTTCCTGAGGCTGGAT |
| IL1B | TGGACCTTCCAGGATGAGGACA | GTTCATCTCGGAGCCTGTAGTG |
| IL-2 | GCGGCATGTTCTGGATTTGACTC | CCACCACAGTTGCTGACTCATC |
| IL-4 | ATCATCGGCATTTTGAACGAGGTC | ACCTTGGAAGCCCTACAGACGA |
| IL-6 | TACCACTTCACAAGTCGGAGGC | CTGCAAGTGCATCATCGTTGTTC |
| IL-10 | CGGGAAGACAATAACTGCACCC | CGGTTAGCAGTATGTTGTCCAGC |
| IL-16 | CACGCAGACTTCATCCTCCACA | AGCTATAGTCCATCCGTGCCTG |
| β-actin | CTATTGGCAACGAGCGGTTCC | CAGCACTGTGTTGGCATAGAGG |
| h-Nos2 | GCTCTACACCTCCAATGTGACC | CTGCCGAGATTTGAGCCTCATG |
| h-Ppp2r3a | CTATGAGGAGCAGTGTGAACGG | GATGTGAGCCATTCTGCACCTC |
| h-β-actin | CACCATTGGCAATGAGCGGTTC | AGGTCTTTGCGGATGTCCACGT |

**Table S1 Sequences of primers used in the study**.

**2.3 CA7 fails to induce protective autophagy**

When mice suffered acute liver injury, the structure and balance of mitochondria in hepatocytes were severely damaged (Fig. S2d). A large number of damaged mitochondria triggered intense autophagy, which in turn aggravated the imbalance of mitochondrial balance. TEM data showed that compared with the control group mice, the number of autophagosomes in the hepatocytes of ALF mice increased significantly, which revealed that D/L-induced autophagic activity in mouse hepatocytes was enhanced, while CA7 pretreatment effectively inhibited excessive autophagic response (Fig. S2a). Consistent with this, IMMUNOFLUORESCENCE staining of mouse tissues showed that compared with the control group, the expression of LC3 A/B in the liver of the model group mice was significantly enhanced, indicating upregulation of autophagy, while CA7 pretreatment weakened the trend of upregulation of autophagy (Fig. S2b). Western blot detection results also confirmed this: D/L can upregulate the expression of LC3B, a key component of autophagosomes, and promote the degradation of autophagic substrate P62 (Fig. S2c, d). In addition, mTOR plays an important role in the regulation of autophagy (1). The enhanced autophagy caused by ALF is accompanied by a decrease in the phosphorylation level of the autophagy inhibitory protein mTOR, while CA7 treatment can significantly restore the phosphorylation level of mTOR in mice. In addition, ALF-induced autophagy activation is not related to Beclin-1 (Fig. S2c, d).

**
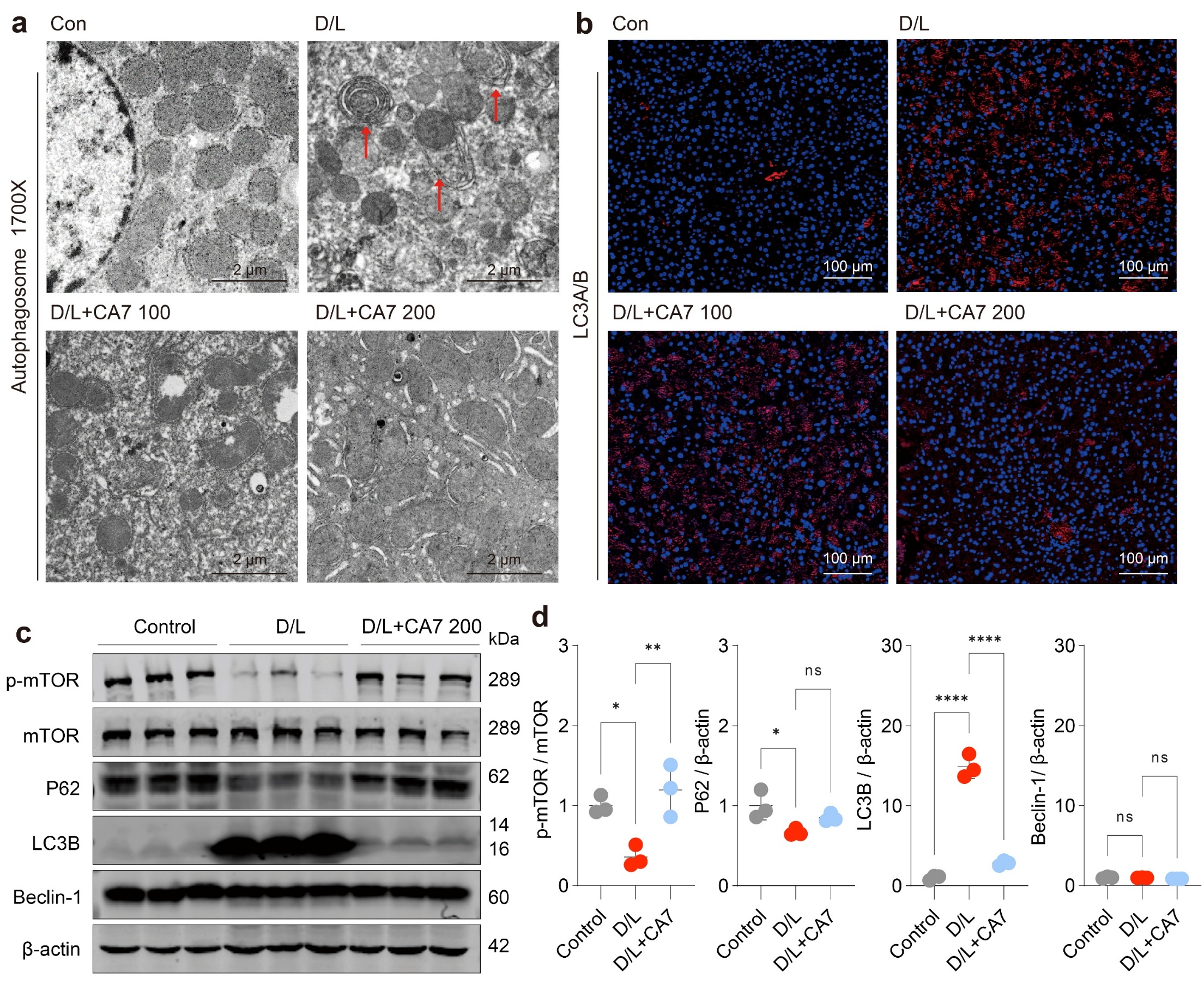
**

**Fig. S2** CA7 inhibits autophagy in hepatocytes. **a** Representative image of autophagosomes in hepatocytes observed by TEM. Scale bar: 2 μm. **b** Representative images of LC3A/B IMMUNOFLUORESCENCE staining in mouse liver tissues. Scale bar: 100 μm. **c** Western blot analysis of protein expression levels of p-mTOR, mTOR, P62, LC3B, and Beclin-1 in mouse liver tissues. β-actin was used as a loading control. **d** Quantitative results of Western blot analysis in **c**. Mean ± SD. One-way ANOVA. n = 3 samples/group.

**2.4 CA7 inhibits oxidative stress in the context of inflammation**

**
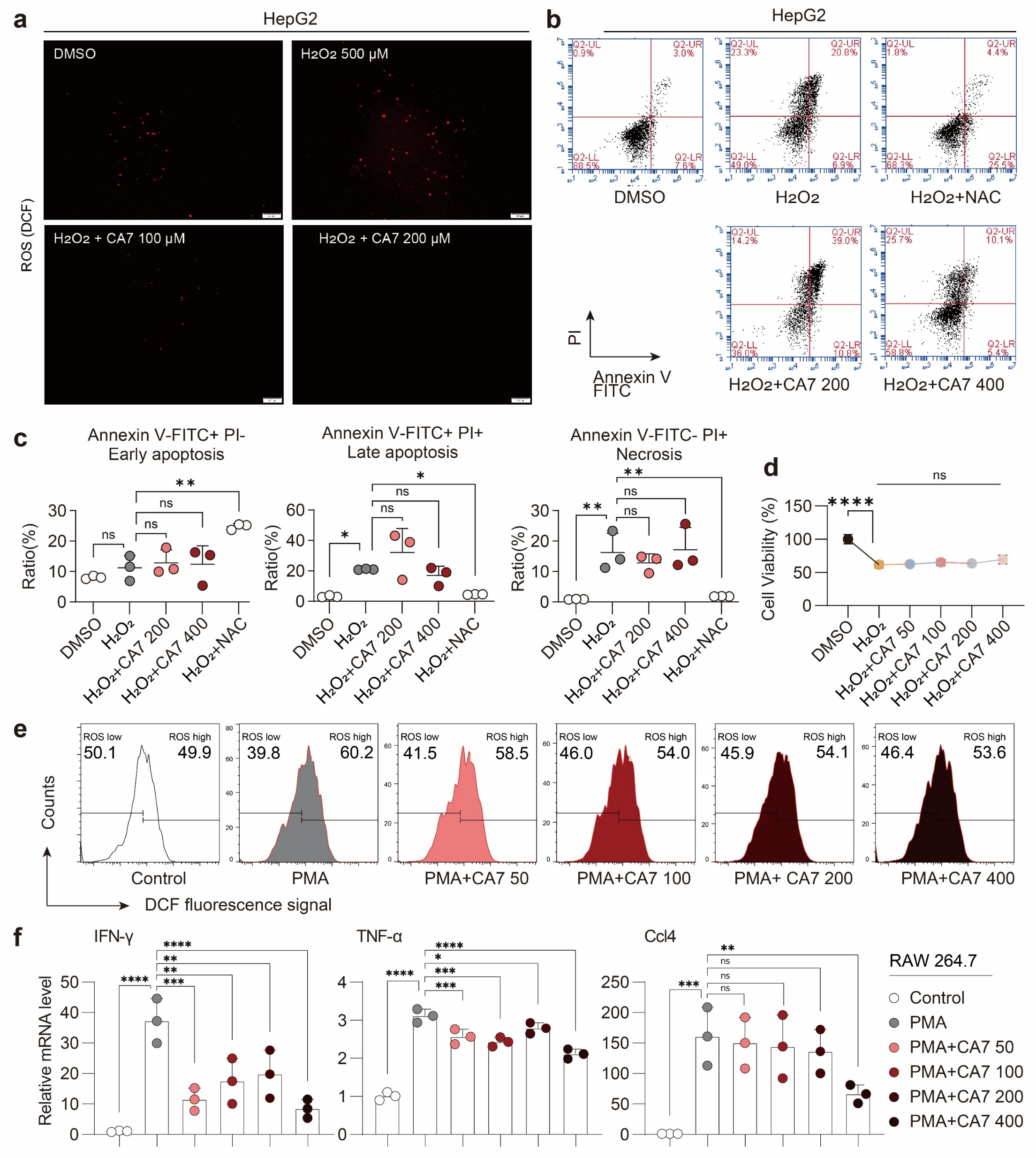
**

**Fig. S3** CA7 Inhibits oxidative stress in RAW 264.7 cells. **a** Representative image of ROS staining in Hep.G2 cells pretreated with CA7 and subsequently exposed to H₂O₂-induced oxidative stress. Scale bar: 50 μm. **b** Flow cytometry analysis reveals that CA7 pretreatment has no significant effect on apoptosis or necrosis in Hep.G2 cells under H₂O₂-induced oxidative stress. Similar results were obtained from three independent experiments. **c** Quantitative analysis of flow cytometry results in **b**. Mean ± SD. One-way ANOVA. n = 3 samples/group. **d** Line graphs of cell viability plotted using CA7 and H₂O₂ when treating HEPG2 cell lines for 24 hours. Mean ± SD. One-way ANOVA. n = 3 samples/group. **e** Flow cytometry analysis of ROS levels in RAW 264.7 cells pretreated with CA7 and stimulated with PMA for 4 hours. **f** qPCR analysis of cytokine mRNA expression levels demonstrates that CA7 inhibits PMA-induced transcription of inflammatory factors. Mean ± SD. One-way ANOVA. n = 3 samples/group.

**2.5 Fig. S4**

**
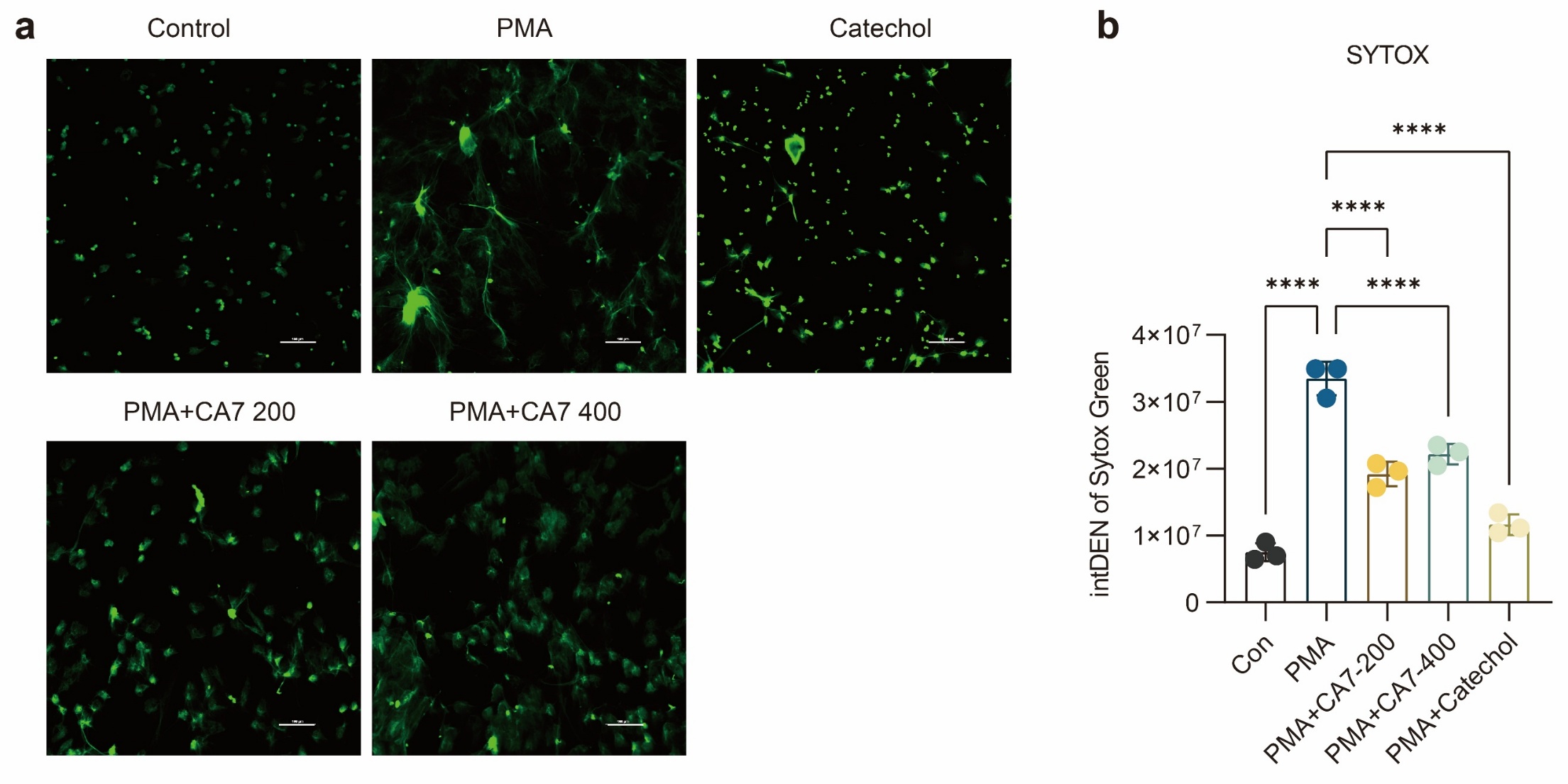
**

**Fig. S4.** CA7 inhibits NET formation. **a** Representative images show reduced NET release upon CA7 treatment. Scale bar: 100 μm. **b** Quantification of SYTOX Green staining intensity (IntDen) in representative images. Mean ± SD. One-way ANOVA. n = 3 samples/group.
